# Co-delivery of gemcitabine and paclitaxel plus NanoCpG empowers chemoimmunotherapy of postoperative “cold” triple-negative breast cancer

**DOI:** 10.1016/j.bioactmat.2023.01.014

**Published:** 2023-01-22

**Authors:** Beibei Guo, Yan Qu, Yinping Sun, Songsong Zhao, Jiandong Yuan, Peizhuo Zhang, Zhiyuan Zhong, Fenghua Meng

**Affiliations:** a Biomedical Polymers Laboratory, College of Chemistry, Chemical Engineering and Materials Science, and State Key Laboratory of Radiation Medicine and Protection, Soochow University, Suzhou, 215123, PR China; b College of Pharmaceutical Sciences, Soochow University, Suzhou, 215123, PR China; c BrightGene Bio-Medical Technology Co., Ltd., Suzhou, 215123, PR China; d GenePharma Co., Ltd., Suzhou, 215123, PR China

**Keywords:** Targeted delivery, Chemoimmunotherapy, Triple-negative breast cancer, Cancer immunotherapy, Combination therapy

## Abstract

Triple-negative breast cancer (TNBC) due to lack of clear target and notorious “cold” tumor microenvironment (TME) is one of the most intractable and lethal malignancies. Tuning “cold” TME into “hot” becomes an emerging therapeutic strategy to TNBC. Herewith, we report that integrin-targeting micellar gemcitabine and paclitaxel (ATN-mG/P, ATN sequence: Ac-PhScNK-NH_2_) cooperating with polymersomal CpG (NanoCpG) effectively “heated up” and treated TNBC. ATN-mG/P exhibited greatly boosted apoptotic activity in 4T1 cells, induced potent immunogenic cell death (ICD), and efficiently stimulated maturation of bone marrow-derived dendritic cells (BMDCs). Remarkably, in a postoperative TNBC model, ATN-mG/P combining with NanoCpG promoted strong anti-cancer immune responses, showing a greatly augmented proportion of mature DCs and CD8^+^ T cells while reduced immune-suppressive myeloid-derived suppressor cells (MDSCs) and regulatory T cells (T_reg_), which led to complete inhibition of lung metastasis and 60% mice tumor-free. The co-delivery of gemcitabine and paclitaxel at desired ratio in combination with NanoCpG provides a unique platform for potent chemoimmunotherapy of “cold” tumors like TNBC.

## Highlights

• Robust small micelles stably co-load gemcitabine and PTX (ATN-mG/P).
• ATN-mG/P facilitates integrin-targeted and synergistic tumor cell ICD and orchestrated APC regulation in TME.
• ATN-mG/P combined with NanoCpG further heats up immune microenvironment.
• The combo brings survival benefits to postoperative TNBC mice with 60% cure.
• The combo provides a strategy to potent chemoimmunotherapy of “cold” tumors.

## Graphical abstract

**Figure:**
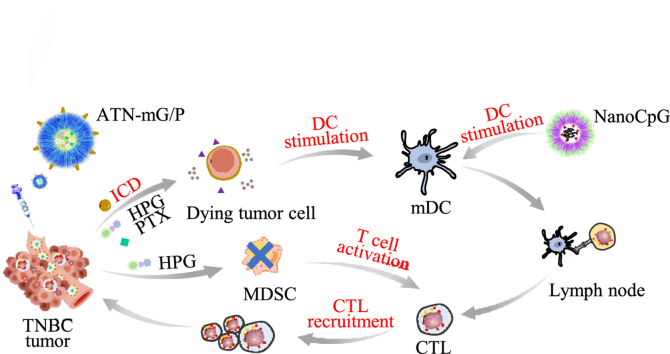

## 1 Introduction

Triple negative breast cancer (TNBC) accounting for 24% of newly diagnosed breast cancer [1] is a most intractable and lethal malignancy. TNBC is highly invasive and prone to brain and lung metastasis, leading to a short survival time of about 13.3 months. The ineffective treatment of TNBC is partly due to lack of targeted molecular drugs [2,3] and its notorious “cold” tumor microenvironment (TME) [3,4]. Immune checkpoint blockade therapy (ICB) in patients with programmed cell death ligand 1 (PD-L1) positive metastatic TNBC displayed a disease control rate of 23.8% and median survival time of 18 months, which did not improve the overall survival compared with chemotherapy [5]. Tuning “cold” TME into “hot” becomes an emerging therapeutic strategy to TNBC [6]. The past years have witnessed various “heating up” strategies *e.g.* by generating tumor antigens with immunogenic cell death (ICD)-inducing chemotherapy, radiotherapy or photodynamic therapy [7,8], trafficking or activating immune cells [9,10], reducing or repolarizing immunosuppressive immune cells [11,12] and remodeling extracellular matrix (ECM) barrier [13]. A couple of chemical drugs like doxorubicin (Dox), paclitaxel (PTX), and oxaliplatin were reported to “heat up” the immune microenvironment by inducing ICD, which led to exposure of calreticulin (CRT) and release of adenosine triphosphate (ATP) and high mobility group box chromosomal protein 1 (HMGB1) [7]. For example, Huang et al. reported the significantly upregulated ICD of 4T1 tumors when treated with Dox (5 mg/kg) and further primed TME in combined with CXC chemokine receptor 4 (CXCR4)-inhibition, resulting in improved anti-PD-L1 therapy [14]. Zhang et al. reported that PTX enhanced the antitumor efficacy of interleukin-12 (IL-12) by spurring ICD of 4T1 tumor cells [15]. The combination therapy with PD-L1 inhibitor atezolizumab and Nab-PTX was recently approved for PD-L1 positive mTNBC [16], though the response rate was only ca. 25%. Further little progress has been made for PD-L1 negative TNBC [17].

The “cold” tumors might also be “heated up” by immunoadjuvants such as toll-like receptors (TLR) agonist (CpG, R848 and R837), indoleamine 2,3-dioxygenase (IDO) inhibitor (NLG919, 1-MT) and stimulator of interferon genes (STING) agonist cyclic dinucleotide [[18], [19], [20]]. Among them, CpG elicits Th1 immune response by inducing the production of type I interferon in immune cells via TLR9 pathway [21]. Mooney et al. reported that an alginate gel loaded with a Dox-iRGD conjugate, granulocyte-macrophage colony stimulating factor (GM-CSF) and CpG, could enhance ICD of tumor cells, increase CD8^+^ T cells, and repolarize tumor-associated macrophages (TAMs) towards M1 phenotype, leading to significantly inhibited TNBC primary tumor and metastases [22]. Ran et al. reported the magnetic nanoparticles loaded CpG which could exert photothermal therapy (PTT) and greatly activate DC maturation to heat up the TME of TNBC [23]. We recently developed a polymersomal CpG (NanoCpG) that greatly boosted the anti-cancer immune responses over free CpG in melanoma and in “cold” orthotopic glioma models [24,25]. It should further be noted that NanoCpG facilitates systemic injection and reduces potential immunogenic toxicity of CpG.

**Scheme 1:**
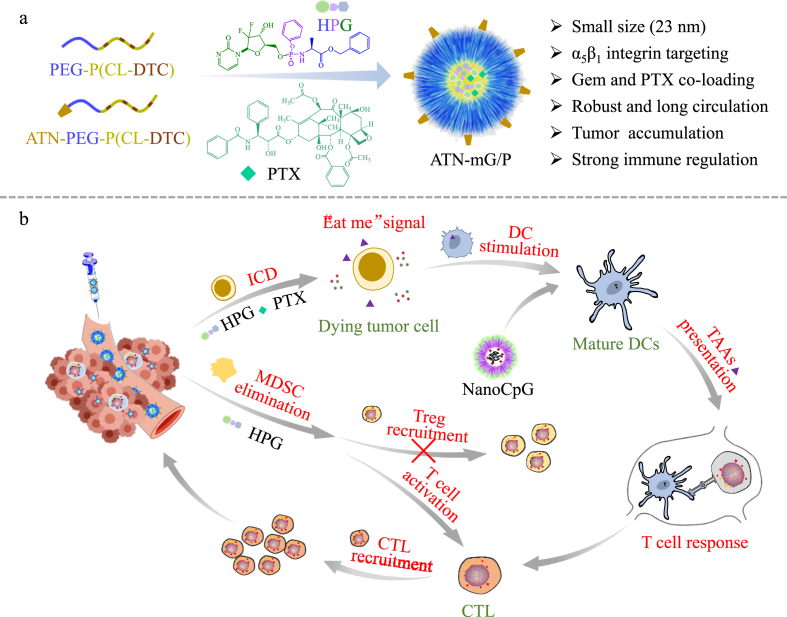
Illustration of the combination therapy of ATN-mG/P and NanoCpG that heat up the immune microenvironment to inhibit progression, recurrence and lung metastasis of 4T1 tumors effectively.

Herewith, we report that integrin-targeting micellar gemcitabine and PTX (ATN-mG/P) cooperating with NanoCpG effectively “heated up” and treated postoperative TNBC mouse model (Scheme 1). ATN-mG/P was designed not only to co-stimulate ICD, but also to reverse immunosuppressive TME in an orchestrated way via activating antigen-presenting cells (APC) by PTX while eliminating MDSCs by Gem. PTX and Gem besides capable of inducing ICD were reported to stimulate APC and reduce MDSCs, respectively [26,27]. To load Gem to micelles, we employed hydrophobic phosphorylated gemcitabine (HPG) prodrug, which has shown better stability and anticancer activity in non-small cell lung tumors than the parent Gem [28]. Remarkably, in a postoperative 4T1 TNBC model, ATN-mG/P plus NanoCpG promoted strong anti-cancer immune responses, leading to complete inhibition of tumor relapse, lung metastasis and 60% mice tumor-free. The co-delivery of Gem and PTX in combination with NanoCpG thus provides a unique platform for potent chemoimmunotherapy of “cold” tumors like TNBC.

## 2 Experimental section

### 2.1 Preparation of mG/P and ATN-mG/P

HPG and PTX were dissolved separately in PEG350 (25 mg/mL) and blended at a molar ratio of 20/1, 10/1 or 5/1. Drug solution (HPG theoretical drug loading: 20 wt.%) was then mixed with 5 μL PEG350 solution of PEG-P(CL-DTC) (200 mg/mL), and PEG350 was added to a final volume of 50 μL. Such mixture was then injected under stirring into 950 μL phosphate buffer (PB, pH 7.4, 10 mM), yielding mG/P with different molar ratios of HPG and PTX. Similarly, for ATN-mG/P with ATN content of 5%, 50 μL PEG350 solution containing 4.75 μL PEG-P(CL-DTC) (200 mg/mL), 2 μL ATN-PEG-P(CL-DTC) (25 mg/mL), HPG/PTX solution and PEG350 were added to 950 μL PB. The size, size distribution and stability of mG/P and ATN-mG/P were determined by dynamic light scattering (DLS). The drug loading and release of mG/P and ATN-mG/P as well as the intact HPG in free HPG, mGem and mG/P (172 μM GEM equiv.) in the presence of cytidine deaminase (CDA) and 10% serum were determined by HPLC (detailed in Supporting Information).

### 2.2 Cytotoxicity studies of mG/P and ATN-mG/P

4T1-luc cells seeded in 96-well plates (1 × 10^3^/well) were added with 20 μL mG/P (Gem/PTX: 20/1, 10/1 or 5/1) with HPG concentration of 0.0017–68.8 μM, or mPTX with PTX concentration of 0.005–12.8 μM. After 48 h incubation, 3-(4,5-dimethylthiazol-2-yl)-2,5-diphenyltetrazolium bromide (MTT) solution (5 mg/mL, 10 μL) was added to incubate for 4 h before washing and addition of 150 μL dimethyl sulfoxide (DMSO) to dissolve the purple formazan produced by living cells. The determination of the cell viability and half-maximal inhibitory concentration (IC_50_) was the same as reported (n = 6) [28].

Combination Index (CI) between two drugs was calculated based on the formula

$$CI=\frac{a}{A}+\frac{b}{B}$$

Here, a and b represent respective the IC_50_ of each drug in mG/P, and A and B represent respective the IC_50_ of each drug in single formulation mGem or mPTX. CI < 1: synergistic effect; CI = 1: additive effect; CI > 1: antagonistic effect.

To investigate the targetability of ATN-mG/P toward 4T1 cells, ATN-mG/P and mG/P at Gem/PTX = 10/1 (HPG conc: 0.0017–68.8 μM) were incubated 4 h with 4T1 cells (1 × 10^3^/well), and the cells were then incubated 44 h with drug-free fresh media. The sample processing and data analysis methods are as described above.

### 2.3 Immunogenic cell death (ICD) of 4T1 cells induced by ATN-mG/P

4T1-luc cells cultured 24 h in 12-well plates (1 × 10^5^/well) were incubated with ATN-mG/P (Gem/PTX: 10/1), mG/P (Gem/PTX: 20/1, 10/1 or 5/1), mGem, or mPTX (HPG: 1 μg/mL, PTX: 0.3 μg/mL, n = 3), taking PBS as control. After 24 h, the culture medium was collected for determining ATP using enhanced ATP assay kit, and the cells for detection of CRT. To determine CRT exposure, these cells were digested, added with αCRT for 1 h, and incubated with Alexa 647-conjugated secondary antibody for 30 min. Between two steps, washing (2 × , cold PBS) was applied. The cells were then measured using flow cytometry and data were analyzed using FlowJo_V10 to determine the CRT expression.

### 2.4 Maturation of BMDCs stimulated by ATN-mG/P

BMDCs cultured in 12-well plates (1 × 10^6^/well) were incubated with ATN-mG/P (Gem/PTX: 10/1), mG/P (Gem/PTX: 20/1, 10/1 or 5/1), mGem, or mPTX (HPG: 1 μg/mL, PTX: 0.3 μg/mL, n = 3), taking PBS as control. After 24 h, the cells were centrifuged, washed and incubated with FITC-αCD11c, APC-αCD80 and PE-αCD86 for 30 min. Then the cells were immediately measured by flow cytometry and analyzed using FlowJo_V10 to quantify mature BMDCs (CD11c^+^CD80^+^CD86^+^ mDCs).

To study the effect of NanoCpG on BMDC maturation, NanoCpG, free G/P (mixture of free HPG and PTX at molar ratio of 10/1), mG/P, ATN-mG/P, mG/P + NanoCpG, and ATN-mG/P + NanoCpG (Gem/PTX = 10/1, HPG: 1 μg/mL, PTX: 0.3 μg/mL, CpG: 0.4 μg/mL) were added to BMDCs. The following treatment and measurement were as described above (n = 3). To study the maturation of BMDCs co-culture with 4T1 cells, BMDCs (1 × 10^6^/well) and 4T1-luc cells (1 × 10^5^/well) were separately cultured in 12-well plates for 24 h. The culture medium of 4T1-luc cells were removed, and BMDCs were added. The two types of cells were then co-incubated with ATN-mG/P (Gem/PTX: 10/1), mG/P (Gem/PTX: 20/1, 10/1 or 5/1), mGem, or mPTX (HPG: 1 μg/mL, PTX: 0.3 μg/mL) for 24 h. The following treatment and measurement were as described above (n = 3).

### 2.5 Therapy of mG/P and ATN-mG/P toward 4T1-luc subcutaneous model

All animal experiments were approved by the Animal Care and Use Committee of Soochow University (P.R. China), and all protocols conformed to the Guide for the Care and Use of Laboratory Animals. 4T1-luc subcutaneous mouse model was built by injecting 50 μL of 4T1-luc cells (3 × 10^5^/mouse with 30 vol.% Matrigel) subcutaneously in the right upper hind leg of Balb/c mice (6 weeks, female). After 7 days the 4T1-luc tumor volume reached ca. 50 mm^3^ (designated as day 0), and the mice were randomly divided into five groups (n = 6): PBS, mPTX (2.25 mg/kg, 2.58 μmol/kg), mGem, mG/P (Gem/PTX: 20/1 or 10/1) at HPG dose of 15 mg/kg (25.8 μmol/kg). On day 0, 2, 4, 6, 8, and 10, drug formulations were intravenously administered into the mice. The tumor volume and body weight were monitored every two days. The survival rates of the mice were recorded and the mice were also considered dead at tumor volume over 2000 mm^3^.

To study the therapeutic efficacy of ATN-mG/P and the immune microenvironment of tumors, ATN-mPTX, ATN-mGem, mG/P, ATN-mG/P (Gem/PTX = 10/1, HPG: 10 mg/kg (17.2 μmol/kg), PTX: 1.5 mg/kg (1.72 μmol/kg)) were i.v. injected into 4T1-luc subcutaneous model, using the same schedule. Four days after last injection (D 14), three mice from each group were euthanized to analyze the infiltration of mDCs and MDSCs in tumor. Tumor tissue was extracted from tumor-bearing mice, ground, centrifuged to obtain single-cell suspension and red blood cells were lysized by red blood cell lysate (ACK). The cells were incubated 30 min at 4 °C with FITC-αCD11c, APC-αCD80 and PE-αCD86 to determine the content of mDCs (CD11c^+^CD80^+^CD86^+^), and with FITC-αCD11b and PE/Cy7-αGr-1 to determine the content of MDSCs (CD11b^+^Gr-1^+^), followed by the flow cytometric analyses.

### 2.6 Chemoimmunotherapy on postoperative 4T1-luc TNBC model

The postoperative recurrent/metastatic 4T1-luc model was established by surgically removing tumor bulk (at volume of 200–300 mm^3^) of subcutaneous 4T1 model as mentioned above. The tumors relapsed quickly and lung metastasis was observed. At seven days after surgery, the relapsed tumor grew to ca.100 mm^3^ (day 0), and the mice were assigned into six groups (n = 6): PBS, free G/P, mG/P, ATN-mG/P, mG/P + NanoCpG, or ATN-mG/P + NanoCpG (Gem/PTX = 10/1, HPG: 15 mg/kg (25.8 μmol/kg), PTX: 2.25 mg/kg (2.58 μmol/kg), CpG: 1.0 mg/kg). On day 0, 2, 4, 6, 8, 10, free G/P, mG/P and ATN-mG/P was injected intravenously. For two combination groups, NanoCpG was injected on day 1, 3, 5. The tumor volume and body weight were monitored every three days. One mouse in each group was euthanized at five days post the last drug-administration to investigate lung metastasis by bioluminescence imaging and H&E staining of lung slices. Other five mice were for observing survival rates. The mice were deemed dead at tumor volume over 2000 mm^3^ or body weight loss over 15%.

### 2.7 Immune analysis of postoperative 4T1 model after treatment

In the postoperative 4T1-luc model, at relapsed tumor volume of ca.100 mm^3^ (day 0), the mice were assigned into five groups (n = 4): PBS, mG/P, ATN-mG/P, mG/P + NanoCpG, or ATN-mG/P + NanoCpG (Gem/PTX = 10/1, HPG: 15 mg/kg (25.8 μmol/kg), PTX: 2.25 mg/kg (2.58 μmol/kg), CpG: 1.0 mg/kg). On day 0, 2, 4 mG/P and ATN-mG/P were i.v. injected. For two combination groups, NanoCpG was injected on day 1, 3, 5. Two days after last injection, the mouse plasma was collected to quantify the concentration of tumor necrosis factor-α (TNF-α), interferon-γ (IFN-γ) and interleukin-10 (IL-10), and the mice were euthanized to study metastasis and immune regulation. The lung was weighed and sliced to count the metastatic nodules. The relapsed tumor was weighed to calculate tumor inhibition rate (TIR). Spleen was weighted and sliced. The lymph node, spleen and tumor were ground, centrifuged to obtain single cell suspensions, and erythrocytes were lysized by ACK. Then the cells were incubated 30 min with corresponding antibodies, i.e. PerpCy5.5-αCD45, FITC-αCD11c, APC-αCD80, PE-αCD86, APC-αCD3, FITC-αCD8, PE-αCD4, Alexa 647-αFoxp3, FITC-αCD11b, Alexa 647-αCD206, PE-αF4-80, PE/Cy7-αGr-1, for determining the contents of mDCs (CD11c^+^CD80^+^CD86^+^), MDSCs (CD11b^+^Gr-1^+^), CD4^+^ T (CD3^+^CD4^+^), CD8^+^ T (CD3^+^CD8^+^), and T_reg_ (CD3^+^CD4^+^FoxP3^+^). The flow cytometry measurements were followed to analyze the infiltration of immune cells in tumor and spleen.

### 2.8 Statistical analysis

Data were presented as mean ± standard deviation. The significant differences among groups were determined using GraphPad Prism 9 by one-way ANOVA (Tukey multiple comparison tests). Survival rate was analyzed by Kaplan-Meier technique with a log-rank (Mantel-Cox) test. *p < 0.05 means significant difference, **p < 0.01, ***p < 0.001 and ****p < 0.0001 mean highly significant difference.

## 3 Results and discussion

### 3.1 Preparation of ATN-mG/P and mG/P

The aim of this study is to tune “cold” TME in TNBC to “hot” therefore enabling efficient immunotherapy, for which we designed integrin-targeting micellar Gem and PTX (ATN-mG/P) to specifically co-deliver both drugs at predetermined ratio to TNBC cells. PTX and Gem both can stimulate ICD. The targeted delivery of Gem and PTX to cancer cells is important to lessen their immune-toxicity. ATN peptide (sequence: Ac-PhScNK-NH_2_) reportedly possessed a high affinity to α_5_β_1_ integrin overexpressed on tumor cells including breast tumor cells and melanoma cells [29,30]. In contrast, cRGD peptide targets α_v_β_3_ and α_v_β_5_ integrins [25,31]. We previously performed a comparative study on ATN and cRGD peptides for targeting 4T1 breast tumor cells, which showed a better targetability of ATN than cRGD (data not shown). In addition to ICD effects, PTX can further activate APCs while Gem can eliminate MDSCs, thereby cooperatively reversing immunosuppressive TME. HPG is a single isomer of Acelarin (Nuc-1031) in which the phosphorous amide bond can be cleaved by intracellular esterase, directly releasing the monophosphate of GEM (dFdCMP) without formation of the inactive form of GEM (dFdU) [28]. The dFdCMP can be converted into diphosphate (dFdCDP) and then triphosphate (dFdCTP) form that can replace deoxycytidine during DNA replication, leading to cell cycle arrest.

**Fig. 1:**
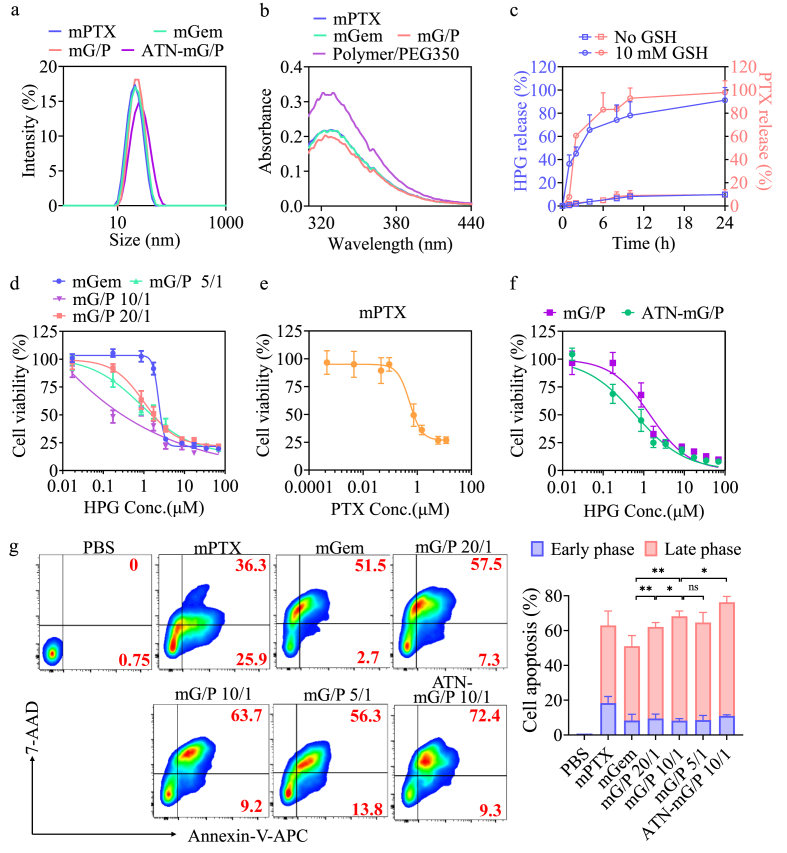
Characterizations of mG/P and ATN-mG/P. (a) Size distribution profiles. (b) The UV absorbance of mGem, mPTX, mG/P and PEG-P(CL-DTC) solution in PEG350. (c) Drug release profiles of mG/P with or without 10 mM GSH at pH 7.4 and 37 °C (n = 3). Cytotoxicity of (d) mG/P, (e) mPTX and (f) ATN-mG/P toward 4T1 cells (n = 6), and (g) cell apoptosis of 4T1 cells at 48 h incubation with ATN-mG/P, mG/P, mGem and mPTX (n = 3). For f, 4T1 cells were cultured with ATN-mG/P and mG/P (Gem/PTX = 10/1) for 4 h and with drug-free medium for 44 h. For g, HPG of 1.7 μM (1 μg/mL) and PTX was at 0.34 μM (0.3 μg/mL) in mPTX. For all ATN-mG/P, Gem/PTX = 10/1. *p < 0.05, **p < 0.01.

Here ATN-mG/P was conveniently prepared via co-self-assembly of a PEG350 solution of 5% ATN-PEG-P(CL-DTC) and 95% PEG-P(CL-DTC) (*M*_n_: 2.0-(1.0–1.0) kg/mol) containing both hydrophobic phosphorylated gemcitabine (HPG) and PTX (Gem/PTX molar ratio = 20/1, 10/1 or 5/1) in an aqueous solution. Similarly, single drug micelles (ATN-mGem and ATN-mPTX) and non-targeted dual drug micelles (mG/P) were prepared as controls. Notably, all micellar formulations had small sizes (19.8–23.2 nm) and particle dispersity index (PDI) (0.08–0.17) (Table 1, Fig. 1a, Fig. S1a). Interestingly, ATN-mG/P and mG/P showed efficient loading of both drugs with efficiencies of 96.6–100% for HPG and 94.9–97.0% for PTX. As comparison, lower efficiencies were observed for single drug formulations (Table 1). The enhanced drug loading in mG/P and ATN-mG/P was possibly due to the presence of π-π stacking or hydrophobic interaction between HPG and PTX. PTX was reported to form strong π-π stacking with other drugs or polymers containing aromatic structure [[32], [33], [34]]. In contrast to the clear solution of ATN-mG/P (Fig. S1b), PTX precipitated quickly without micelles in otherwise the same condition. ATN-mG/P and mG/P could maintain colloidal stability for at least one week at room temperature (Fig. S1c) and in PB containing 10% FBS for 24 h (Fig. S1d). The stability was mostly owing to the ring-opening of dithiolanes to form disulfide-crosslinked micellar core as revealed by decreased UV absorbance of dithiolanes (Fig. 1b). In contrast to a minimal drug release (<20%) from mG/P in 24 h at pH 7.4, over 90% of HPG and PTX were released under 10 mM GSH (Fig. 1c). Notably, for mGem, over 60% HPG was discharged in 8 h at pH 7.4 in the absence of GSH (Fig. S1e). These results indicate that the interactions like π-π stacking between HPG and PTX could inhibit drug leakage and enhance the stability of mG/P.

**Table 1:** Characterization of HPG and PTX co-loaded micelle mG/P and ATN-mG/P.^a^.

Nanoparticles	Size^b^ (nm)	PDI	HPG loading^c^		PTX loading^c^	
			loading content (*wt.*%)	loading efficiency (%)	loading content (*wt.*%)	loading efficiency (%)
mG/P 20/1	20.2	0.17	8.9	97.7	0.66	97.0
mG/P 10/1	22.5	0.17	9.1	100.0	1.29	94.9
ATN-mG/P 10/1	23.2	0.15	8.8	96.6	0.66	96.8
mGem	22.0	0.12	6.1	65.6	–	–
ATN-mGem	23.2	0.11	6.2	68.1	–	–
mPTX	19.8	0.08	–	–	5.4	79.4
ATN-mPTX	20.1	0.12	–	–	5.2	76.5

a At theoretical HPG loading content 9.1 *wt.*%.

b Determined by DLS in PB (pH 7.4, 10 mM).

c Determined by HPLC.

### *3.2* *In vitro* anti-tumor efficacy of mG/P and ATN-mG/P

MTT assay was used to evaluate the inhibitory effect of mG/P and ATN-mG/P toward 4T1 cells. Fig. 1d displays that mG/P at Gem/PTX molar ratios of 20/1, 10/1 or 5/1 had considerably lowered IC_50_ (μM) of 2.3/0.10, 0.4/0.07 and 1.8/0.16 (corresponding to IC_50_ of HPG and PTX), respectively, compared to those of mGem and mPTX (3.8 and 1.4 μM) (Fig. 1d and e). The combination indexes (CI) [35,36] of HPG and PTX in co-loaded micelles mG/P were all below 1, with the lowest CI (0.16) at Gem/PTX of 10/1 (Table S1), pointing to a strong synergistic effect between HPG and PTX within mG/P.

Our previous work revealed that ATN-mPTX with 5% ATN performed the best *in vitro* and *in vivo* [37]. Here ATN-mG/P with 5% ATN was prepared and investigated for targeting to breast tumor. The results displayed a further 2.3-fold lower IC_50_ of ATN-mPTX than non-targeting mG/P at the same Gem/PTX of 10/1 (Fig. 1f), verifying the targetability to 4T1 cells. If not stated otherwise, ATN-mG/P denoted an ATN content of 5% and Gem/PTX = 10/1. The cell apoptosis analyses demonstrated that mG/P at Gem/PTX = 10/1 provoked the highest apoptosis among mG/P with other ratios (*p) and single drugs (*p), and ATN-mG/P further improved the apoptotic activity (*p) (Fig. 1g). The cell cycle study showed that mG/P at low concentrations (HPG: 0.1 μg/mL; PTX: 0.03 μg/mL) severely disturbed cell cycle with both enormous S and G2/M arrest (Fig. S2), in sharp contrast to mild cell cycle arrest for mPTX and mGem, revealing the synergistic effect. It was generally reported that PTX typically caused G2/M arrest [38] and Gem caused S arrest [28] at a couple of magnitude higher concentrations. Besides significantly enhanced cell cycle arrest, increased stability of GEM inside cells against CDA degradability by PTX may also contribute to the synergistic effect of mG/P. Previous study has shown that HPG and the loading by micelles could both enhance the resistance of enzymatic degradation [28]. Fig. S3 indicated that at 4 h incubation in the presence of CDA and 10% FBS, the intact HPG in mG/P group was about 7-fold that of HPG-loaded micelles mGem, supporting that PTX can protect HPG from degradation.

### 3.3 Immunogenic cell death (ICD) induced by mG/P and ATN-mG/P

**Fig. 2:**
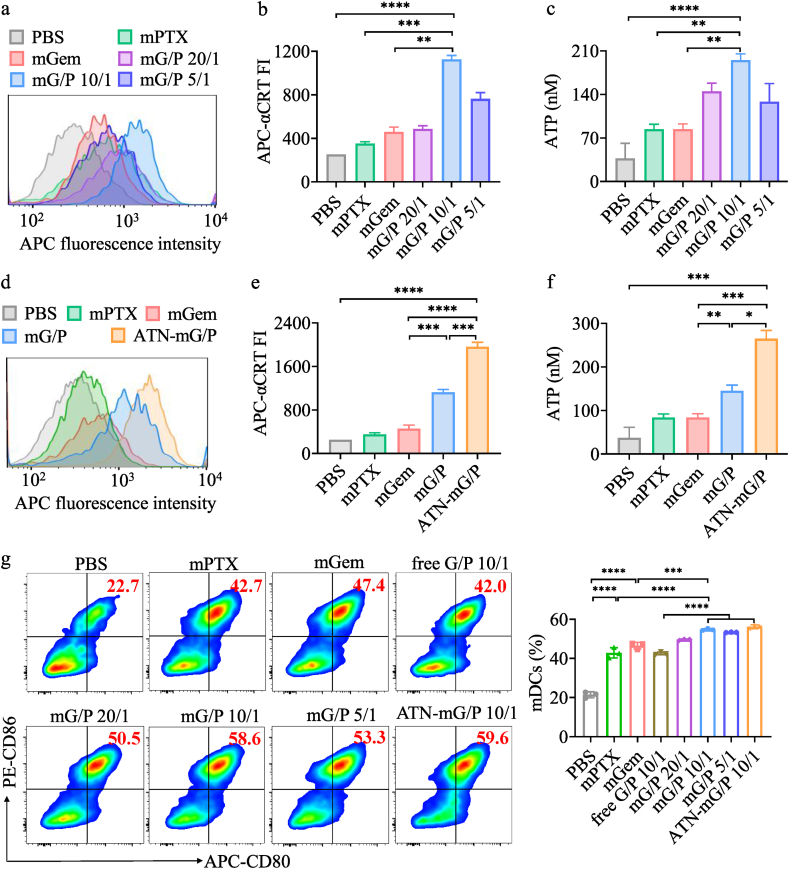
The effect of mG/P and ATN-mG/P on 4T1 tumor cells and BMDCs at 24 h incubation (n = 3). Expression of (a,b) CRT and (c) ATP of 4T1 cells treated with mG/P (Gem/PTX of 20/1, 10/1, 5/1). Expression of (d,e) CRT and (f) ATP of 4T1 cells treated with ATN-mG/P (Gem/PTX of 10/1). (g) BMDC maturation (CD80^+^CD86^+^ mDCs) stimulated by mG/P and ATN-mG/P measured using flow cytometry. HPG dose: 1 μg/mL (1.7 μM). For mPTX control, PTX dose was 0.3 μg/mL (0.34 μM). *p < 0.05, **p < 0.01, ***p < 0.001, ****p < 0.0001.

Certain chemodrugs can modulate the microenvironment of cold tumors by providing tumor antigens via inducing ICD of tumor cells [7,39] or by stimulating APCs to promote the cancer-immune cycle. PTX and Gem are two interesting candidates with potential synergistic effects when co-loaded into robust micelles. To evaluate the ICD and APC stimulating effect, mG/P and ATN-mG/P at low concentrations (HPG: 1.7 μM, PTX: 0.34 μM) were investigated using flow cytometry to detect the production of CRT and ATP, which are typical markers of ICD. The results revealed that mPTX and mGem caused only slight increase of CRT and ATP, while mG/P at Gem/PTX = 10/1 induced marked production of CRT and ATP, which was noticeably higher than single drug micelles and mG/P at Gem/PTX = 5/1 or 20/1 (Fig. 2a–c), signifying a vital role of Gem/PTX ratio in micelles. Of note, ATN-mG/P stimulated further significantly more secretion of CRT and ATP by 4T1 cells than mG/P (***p, *p) (Fig. 2d–f).

We investigated the stimulation of dendritic cells (DCs), the most important APCs, by ATN-mG/P. Flow cytometry results demonstrated that mPTX and mGem at low concentrations could both greatly stimulate BMDCs to mature (CD80^+^CD86^+^ mDCs) (****p). It is reported that low-dose PTX can promote the maturation and proliferation of DC cells [40], due to the fact that PTX could regulate the proliferation and polarization of APCs via TLR4 pathway [26]. However, little was reported on DC stimulation by Gem, except its ability of eliminating MDSCs [27,41]. Our results displayed that mGem stimulated BMDC maturation greatly to 47.4%, probably due to the up-regulation of heat shock protein 70 [42]. Remarkably, mG/P at Gem/PTX of 10/1 further enhanced DC maturation to 58.6% compared with mGem (***p), which was also significantly higher than mixture of free PTX and free HPG (free G/P, ****p) (Fig. 2g), illustrating a synergistic effect of mG/P on stimulating DC maturation. Notably, mG/P and ATN-mG/P showed similar stimulation of BMDCs, likely due to little effect of ATN on the endocytosis of micellar formulations by BMDCs.

It is known that tumor antigens induced by ICD can serve as “eat-me” signal to promote DC maturation and antigen presentation to T cells, leading to tumor-specific T-cell response. To simulate tumor microenvironment, we further studied the effect of mG/P and ATN-mG/P on DC maturation when co-cultured with 4T1 cells (Fig. S4a). The results disposed that the DC stimulation by mPTX and mGem was quite low, probably due to their preferential endocytosis by 4T1 cells (Fig. S4b). mG/P (Gem/PTX of 10/1) exhibited the highest DC maturation among all non-targeted formulations, and ATN-mG/P induced further more enhanced proportion of mDCs (40.3%, ***p) (Fig. S4c). This enhanced DC maturation is likely associated with effective co-delivery of Gem and PTX, which improves direct DC stimulation as well as indirect stimulation from tumor antigens produced by ICD of 4T1 cells.

### 3.4 Antitumor efficacy of mG/P and ATN-mG/P on 4T1 tumor bearing mice

**Fig. 3:**
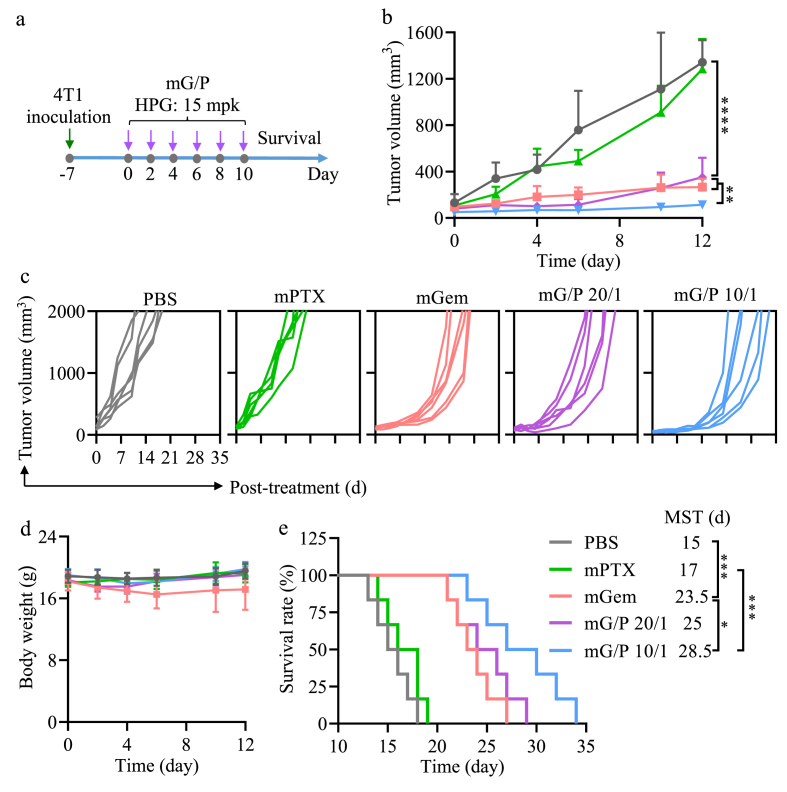
The therapy of mG/P on 4T1 tumor bearing mice (n = 6). (a) Treatment schedule. The mice were intravenously injected on day 0, 2, 4, 6, 8, 10 at mG/P (Gem/PTX of 20/1 (HPG:15 mpk (25.8 μmol/kg), PTX: 1.13 mpk (1.29 μmol/kg)) and 10/1 (HPG:15 mpk (25.8 μmol/kg), PTX: 2.25 mpk (2.58 μmol/kg)) and mGem (HPG: 15 mpk, 25.8 μmol/kg). mPTX (PTX: 2.25 mpk, 2.58 μmol/kg) was as control. (b) Tumor volume, (c) individual tumor growth curves, (d) body weight, and (e) survival curves of the mice. *p < 0.05, **p < 0.01, ***p < 0.001, ****p < 0.0001.

Encouraged by the promising results in anti-TNBC cells and DC stimulation, we investigated the antitumor efficacy of mG/P and ATN-mG/P in murine 4T1-luc TNBC mouse model. Seven days after subcutaneous inoculation of 4T1-luc cells, tumors grew to average volume of ca. 50 mm^3^, and the mice were randomly grouped (designated as day 0) and intravenously injected with mG/P (Gem/PTX of 20/1 or 10/1), mGem (HPG: 15 mpk, 25.8 μmol/kg), or mPTX (PTX: 2.25 mpk, 2.58 μmol/kg) every two days (mpk: mg/kg) (Fig. 3a). The results illustrated that mG/P and mGem treatment effectively retarded tumor progression (****p) (Fig. 3b and c). In contrast, mPTX had little inhibitory effect due to a low dose applied. We reported previously that mPTX could suppress 4T1 tumor growth at 7.5 mpk [37]. mG/P at Gem/PTX of 10/1 had the best tumor inhibition and significantly better than mGem and mG/P at Gem/PTX of 20/1 (**p) (Fig. 3b and c). Except that mGem induced slight body weight loss, all other groups exhibited little body weight change (Fig. 3d). Fig. 3e displays that mG/P at Gem/PTX = 10/1 meaningfully prolonged the median survival time (MST) to 28.5 d, which was significantly better than those of mPTX and mGem (***p and *p).

**Fig. 4:**
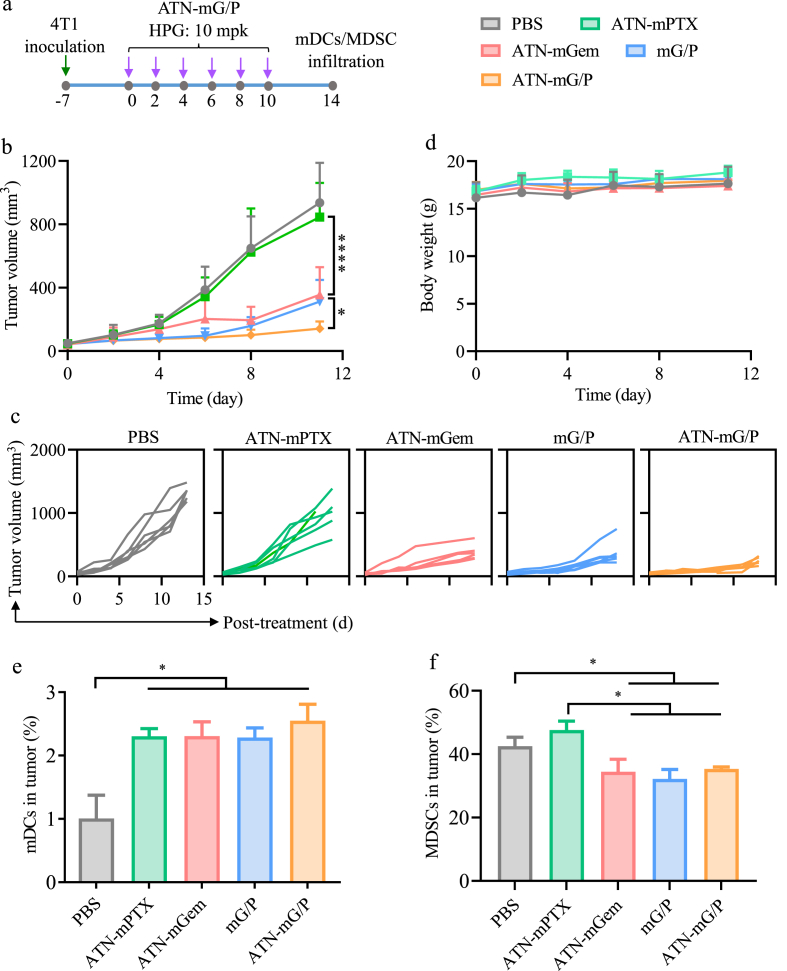
The therapy of ATN-mG/P on 4T1 tumor bearing mice (n = 6). (a) Treatment schedule. The mice were intravenously injected on day 0, 2, 4, 6, 8, 10 with ATN-mGem, mG/P and ATN-mG/P (Gem/PTX of 10/1, HPG: 10 mpk (17.2 μmol/kg), PTX: 1.5 mpk (1.72 μmol/kg)). ATN-mPTX was as control. (b) Tumor volume, (c) individual tumor growth curves, (d) body weight of the mice (n = 6). The proportions of (e) mDCs and (f) MDSCs infiltrated in tumors (on day 14) of the mice (n = 3). *p < 0.05, ****p < 0.0001.

To further investigate the *in vivo* active-targeting effect and the regulation of TME of 4T1-luc mice, ATN-mG/P and mG/P were intravenously injected using the same schedule. Targeted micelles containing only one drug, ATN-mPTX and ATN-mGem, were used as controls (Fig. 4a). Fig. 4b and c shows that ATN-mG/P induced significantly better tumor inhibition than both non-targeting mG/P and ATN-mGem (*p), while ATN-mPTX had practically no tumor inhibition. All treatments did not cause body weight loss (Fig. 4d). It is known that TNBC is a highly immunosuppressive tumor with ca. 40% MDSCs yielding low reponse rate of TNBC patients to immune therapy [17,43]. To assess the effect of ATN-mG/P on tumor immue microenvironment, on day 14 the mice were sacrificed to analyze the infiltration of MDSCs and DCs in 4T1 tumors. Flow cytometry results showed that the proportion of mature DCs in tumors was enhanced by 2.2–2.5 folds with all four micellar drugs (*p) (Fig. 4e). MDSC proportion in tumors of PBS group was as high as 42% (Fig. 4f), supporting a highly immunosuppressive nature of TNBC tumors [43]. The treatment with mG/P, ATN-mG/P or ATN-mGem greatly reduced MDSCs in tumors (*p), which was ascribable to MDSC elimination effect of Gem [27]. ATN-mPTX alone instead somewhat up-regulated MDSC content. The study on size changes and HPG release of mG/P under different GSH concentrations revealed fast response of mG/P to 10 mM GSH (intracelluar reductive condition) while obvious reponse was also observed at 0.1 mM GSH (TME reductive condition) in 72 h (Fig. S5), indicating that HPG can be released from mG/P in TME for MDSC elimination.

### 3.5 Therapeutic activity of ATN-mG/P on postoperative recurrent/metastatic 4T1 model

**Fig. 5:**
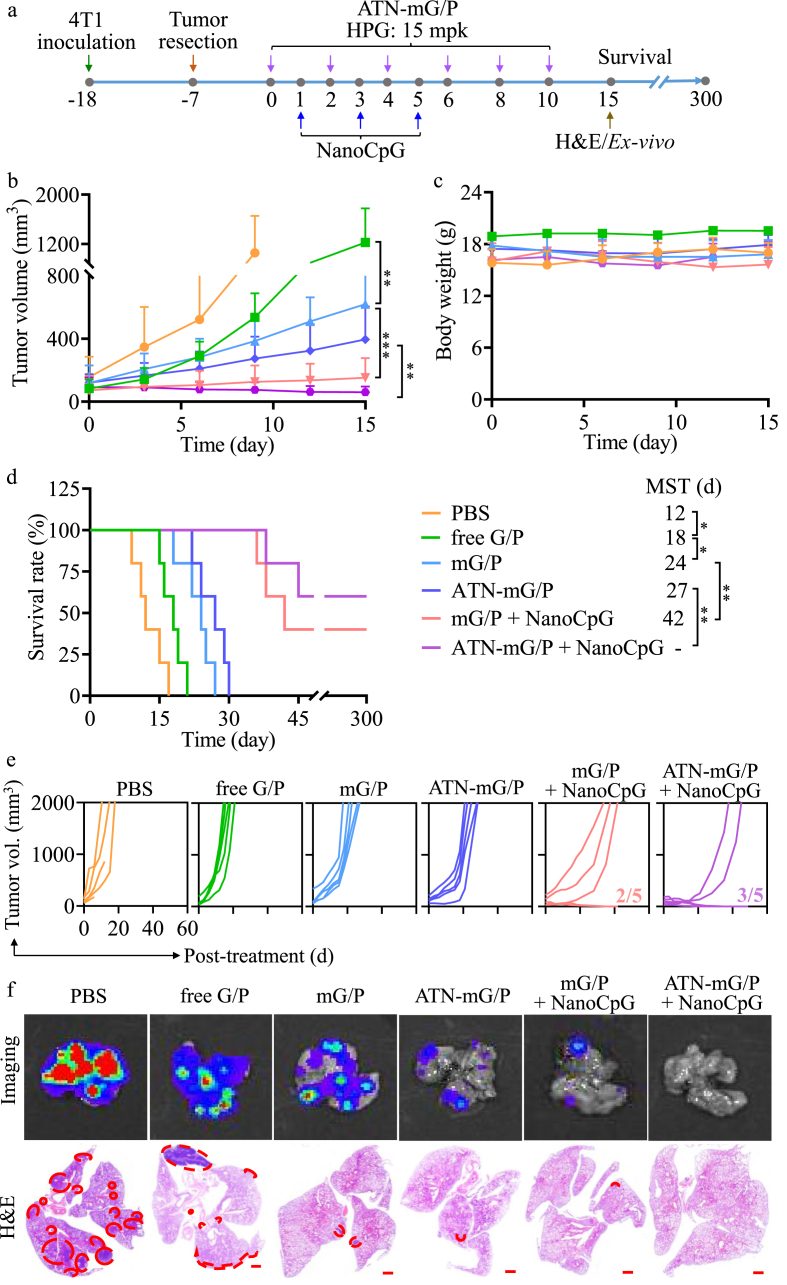
Chemoimmunetherapy of mG/P and ATN-mG/P (Gem/PTX = 10/1) combined with NanoCpG on recurrent/metastatic 4T1-luc mice. (a) Workflow. ATN-mG/P and mG/P (Gem/PTX = 10/1) were i.v. injected on day 0, 2, 4, 6, 8, 10 (HPG: 15 mpk (25.8 μmol/kg), PTX: 2.25 mpk (2.58 μmol/kg)) and NanoCpG on day 1, 3, 5 (CpG: 1 mpk). (b) Tumor volume (n = 6), (c) body weight (n = 6), and (d) survival rates of the mice (n = 5). (e) Individual tumor growth curve (n = 5). (f) *Ex-vivo* fluorescent and H&E images (scale bars: 1000 μm) of whole lung scans on day 15. Red circles show the pulmonary nodules caused by metastasis of 4T1-luc cells. *p < 0.05, **p < 0.01,***p < 0.001.

Encouraged by their tumor inhibition and immune microenvironment regulation effect, we challenged the therapeutic effects of mG/P and ATN-mG/P in postoperative recurrent/metastatic TNBC model. The mouse model was established by surgically removing tumor bulks at eleven days after inoculation of 4T1 cells (Fig. 5a). The recurrent tumors grew much faster than the primary tumors (Fig. 5b). Despite of no body weight loss during the administration period (Fig. 5c), the MST of PBS group was reduced to 12 d (Fig. 5d). Interestingly, the growth of recurrent tumors was drastically restrained by mG/P and ATN-mG/P (**p) (Fig. 5b), and the MST was prolonged to 24 d (mG/P) and 27 d (ATN-mG/P) (Fig. 5d). The individual tumor growth curves of ATN-mG/P showed that tumors finally relapsed again and grew rapidly (Fig. 5e). Lung metastasis frequently occurred in recurrent tumors and accounted for 36.9% for recurrent TNBC patients, leading to a low 5-year survival rate [44,45]. Of note, mG/P and ATN-mG/P drastically reduced tumor nodules in the lungs compared to PBS group that had massive tumor metastasis (Fig. 5f). It is noticed that free G/P mixture was inferior to mG/P and ATN-mG/P in inhibtion of both tumor growth (**p) and lung metastisis.

### 3.6 Chemoimmunotherapy of postopertive recurrent/metastatic TNBC model

To further improve the anti-metastasis capability and survival benefit of ATN-mG/P toward recurrent TNBC model, we explored the chemoimmunotherapy by combining ATN-mG/P with NanoCpG. CpG is a TLR9 agonist, and has been widely used as immunoadjuvant for cancer immunotherapy in preclinical studies and clinical trials [46,47]. CpG was frequently applied intra-tumorally [48], however it is not applicable for inaccessible tumors and also associated with immunogenic toxicities. Besides, CpG has inefficient cellular uptake and fast degradation *in vivo*. Recently we have developed a NanoCpG, CpG-loaded polymersomes, which can be applied intravenously for treating glioma and melanoma in adjuvant with low-dose X-ray, proteins or oncolytic peptide [24,25]. Here, NanoCpG from PEG-PTMC based disulfide-crosslinked polymersomes showed robust loading of CpG ODN and small sizes (50 nm, PDI 0.10) as an adjuvant for immune therapy. Flow cytometric results exhibited that the combination of NanoCpG with mG/P and ATN-mG/P further potently stimulated BMDC maturation to 77.0% and 85.6% (****p), respectively (Fig. S6).

The chemoimmunotherapy using ATN-mG/P or mG/P combined with NanoCpG toward postoperative TNBC model was explored (Fig. 5a). It is known that CpG was not effective in treating 4T1 tumors. Notably, ATN-mG/P and mG/P combining with i.v. injection of NanoCpG (1 mpk) led to markedly enhanced suppression of tumor recurrence and lung metastasis. The tumor growth was halted by mG/P + NanoCpG, and shrinkage of tumors was even observed for ATN-mG/P + NanoCpG group (Fig. 5b). Remarkably, mG/P and ATN-mG/P in combination with NanoCpG led to significantly extended MST (**p), in which 2/5 and 3/5 mice were completely cured with tumor-free, respectively (Fig. 5d and e). It is further noted that for non-cured mice, mG/P and ANT-mG/P in combination with NanoCpG achieved effective suppression and elimination of lung metastasis (Fig. 5f). In comparison, the chemo-immunotherapy of Dox-liposomes and αPD-1 did not prevent lung metastasis of 4T1 tumors, and only further combination with losartan as stroma-depleting agent could improve α-PD1 efficacy [49]. The MST of 4T1 tumor-bearing mice received chemo-immunotherapy of nanomedicines of PTX and STING agonists ADU-S100 was only 32 days with partially inhibited lung metastasis [37].

### 3.7 Analysis of immune cell infiltration and cytokine secretion

**Fig. 6:**
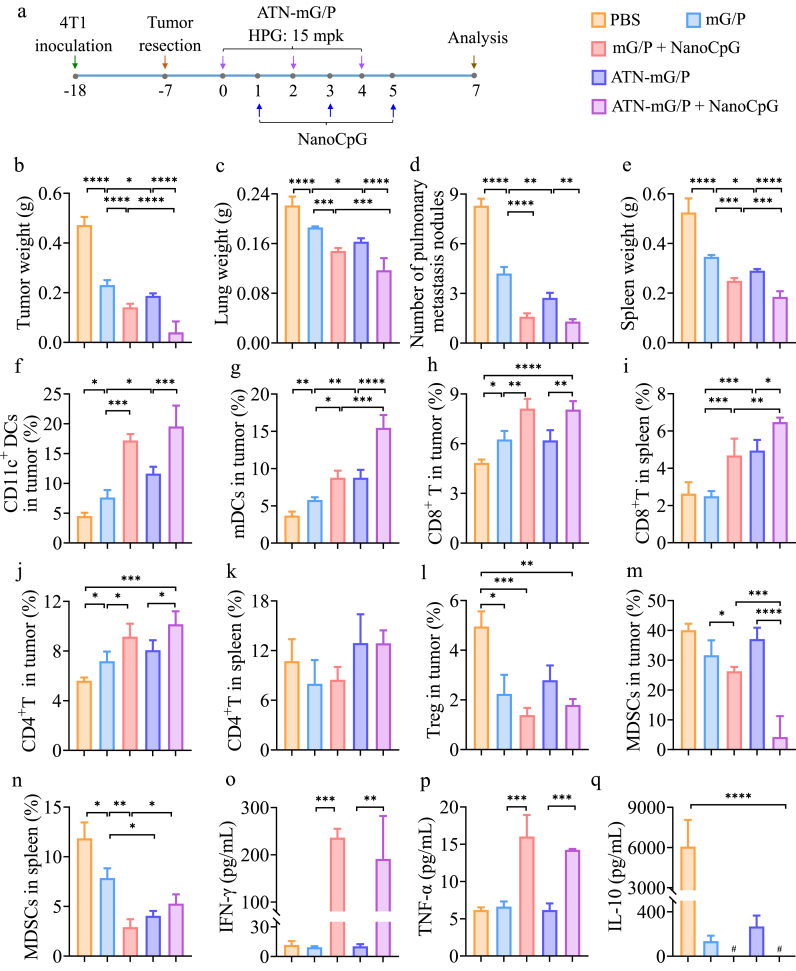
Analysis of tumor environment of postoperative TNBC mice after chemoimmunotherapy (n = 4). (a) Workflow. ATN-mG/P and mG/P (Gem/PTX of 10/1) were i.v. injected on day 0, 2, 4 (HPG: 15 mpk (25.8 μmol/kg), PTX: 2.25 mpk (2.58 μmol/kg)) and NanoCpG on day 1, 3, 5 (CpG: 1 mpk). (b) Tumor weight, (c) lung weight, (d) number of lung metastasis nodules and (e) spleen weight of the mice. The proportions of (f) CD11c^+^ DCs and (g) CD11c^+^CD80^+^CD86^+^ mDCs infiltrated in tumor. The proportions of CD8^+^ T cells in (h) spleen and (i) tumor. The proportions of CD4^+^ T cells in (j) spleen and (k) tumor. (l) The proportions of T_reg_ in tumor. The proportions of MDSCs in (m) tumor and (n) spleen. The plasma concetration of (o) IFN-γ, (p) TNF-α and (q) IL-10 determined by EILSA. # means below the detection limit (8 pg/mL). *p < 0.05, **p < 0.01, ***p < 0.001, ****p < 0.0001.

To better understand the effect of chemoimmunetherapy of mG/P + NanoCpG and ATN-mG/P + NanoCpG on tumors and TME regulation of postoperrative TNBC model, we investigated the immunological responses (immune cells and cytokines) of the mice (Fig. 6a). On 48 h after the third injection of NanoCpG, the mass of recurrent tumors of PBS group (0.5 g) was considerably abridged by all four formulations with ATN-mG/P + NanoCpG (0.05 g) being the smallest (Fig. 6b). The mass of lungs with metastatic tumors followed the same trend showing ATN-mG/P + NanoCpG (0.12 g) group with similar that of healthy mice (Fig. 6c). As seen from H&E staining images of whole lung scans, pulmonary metastatic nodules of PBS group (about 8 nodules) was drastically reduced by both combinaiton groups (Fig. 6d). The spleen of PBS group was enlarged enormously, and this splenomegaly occurs typically during the onset of TNBC and is caused by large infiltration of immune cells but not normally activated since spleen is a major immune organ. Splenomegaly was prevented by ATN-mG/P + NanoCpG, giving similar mass to that of healthy mice (0.18 g) (Fig. 6e).

The great therapeutic effecacy of ATN-mG/P + NanoCpG treatment was manifested by significantly boosted infiltration of total CD11c^+^ DCs and CD11c^+^CD80^+^CD86^+^ mDCs in recurrent tumors as compared to all other groups (Fig. 6f and g). The higher tumor accumulation of ATN-mG/P together with low dose of PTX and CpG could lead to promoted DC recuitment and maturation activated via TLR4 and TLR9 pathways, respectively. Consequently, the highly promoted mDCs enabled more T cell recruitment and antigen-presentation, resulting in a sturdy tumor-specific T cell response. T cells, especially CD8^+^ cytotoxic T cells (CTL) can attack and kill tumor cells directly. Flow cytometry analysis results confirmed that CD8^+^ T and CD4^+^ T cells in spleen and tumor of ATN-mG/P + NanoCpG group were all higher than those in monotherapy groups (Fig. 6h, i, j, k). In particular, the content of immunosuppressive regulatory T cells (T_reg_) in CD4^+^ T cells that weaken T cell activity in TME was drastically decreased by all treatments (Fig. 6i). The MDSC infiltration in recurrent tumor and spleen was about 40% and 12%, respectively. The four treatments could all lessen MDSC infiltration in recurrent tumor and spleen (Fig. 6m, n). Remarkably, tumor MDSC infiltration was reduced tremendously by ATN-mG/P + NanoCpG to <4%. Suzuki et al. also reported that Gem selectively eliminated MDSCs in tumors concomitantly with an enhanced antitumor activity of CD8^+^ T cells and activated natural killer (NK) cells, with no reduction in typical immune cells [27].

Interestingly, ELISA assay results showed that plasma concentrations of IFN-γ and TNF-α were signifacantly stimulated by mG/P + NanoCpG and ATN-mG/P + NanoCpG, compared with mG/P and ATN-mG/P (***p, **p) (Fig. 6o, p). IFN-γ and TNF-α are typical pro-inflammatory cytokines and can improve the activity of NK, DC and CTLs. The mice receiving mG/P + NanoCpG or ATN-mG/P + NanoCpG displayed high plasma IFN-γ levels. Importantly, no acute systemic inflammatory symptoms such as behavioral abnormalities or weight loss were observed, and the lung slices of these two groups did not show monocyte/neutrophil infiltration, alveolar wall thickening, and septal edema (Fig. S7), indicating that mG/P + NanoCpG or ATN-mG/P + NanoCpG does not cause cytokine storm. It is interesting to note that IL-10 was significantly decreased by all four groups, and concentrations of IL-10 of the two combo groups were below detection limit of ELISA kit (<8 pg/mL), confirming the positive correlation of IL-10 with MDSCs (Fig. 6q) [50]. The above results collectively proved that ATN-mG/P + NanoCpG produced a strong tumor-specific immune response, thus achieving excellent therapeutic efficacy on postoperative recurrent/metasic TNBC model.

## 4 Conclusion

We have demonstrated that integrin-targeting micellar gemcitabine and paclitaxel (ATN-mG/P) cooperating with polymersomal CpG (NanoCpG) can effectively heat up “cold” tumor microenvironment, resulting in potent chemoimmunotherapy of postoperative recurrent/metastatic TNBC model. Remarkably, 3/5 4T1-bearing mice have been cured by ATN-mG/P + NanoCpG group. This exceptional chemo-immunotherapeutic efficacy is likely a result of a collective effect of effective recruitment and activation of DCs, good antigen-presenting to CD8^+^ and CD4^+^ T cells, inclined production of TNF-α and IFN-γ, as well as decrease of immune-suppressive MDSCs, T_reg_ and IL-10. This co-delivery of gemcitabine and paclitaxel in combination with NanoCpG adjuvant seems to be a particularly powerful strategy to improvement of the chemoimmunotherapy of “cold” tumors like TNBC.

## Ethics approval and consent to participate

All animal experiments were approved by the Animal Care and Use Committee of Soochow University (P.R. China), and all protocols conformed to the Guide for the Care and Use of Laboratory Animals.

## CRediT authorship contribution statement

**Beibei Guo:** Formal analysis, Data curation, Writing – original draft. **Yan Qu:** Formal analysis, Data curation. **Yinping Sun:** Formal analysis, Data curation. **Songsong Zhao:** Formal analysis, Data curation. **Zhiyuan Zhong:** Conceptualization, Supervision. **Fenghua Meng:** Conceptualization, Supervision, Writing – review & editing.

## Declaration of competing interest

The authors declare that they have no known competing financial interests or personal relationships that could have appeared to influence the work reported in this paper.
