# Supplementary material for: Co-delivery of gemcitabine and paclitaxel plus NanoCpG empowers chemoimmunotherapy of postoperative “cold” triple-negative breast cancer
Source: Bioact Mater. 2023 Jan 22;25:61–72. doi: 10.1016/j.bioactmat.2023.01.014 (PMC9879764; doi:10.1016/j.bioactmat.2023.01.014)
Supplement: Multimedia component 1 [file mmc1.docx]

*Supporting information for*

**Co-delivery of gemcitabine and paclitaxel plus NanoCpG empowers chemoimmunotherapy of postoperative “cold” triple-negative breast cancer**

Beibei Guo^1,2^, Yan Qu^1^, Yinping Sun^1^, Songsong Zhao^1^, Jiandong Yuan^3^, Peizhuo Zhang^4^, and Zhiyuan Zhong^1,2,*^, Fenghua Meng^1,2,*^

^1^ *Biomedical Polymers Laboratory, College of Chemistry, Chemical Engineering and Materials Science, and State Key Laboratory of Radiation Medicine and Protection, Soochow University, Suzhou, 215123, P. R. China*

^2^ *College of Pharmaceutical Sciences, Soochow University, Suzhou, 215123, P. R. China*

^3^ *BrightGene Bio-Medical Technology Co., Ltd., Suzhou, 215123, P. R. China*

^4^ *GenePharma Co., Ltd., Suzhou, 215123, P. R. China*

*Corresponding authors. Tel/Fax: +86-512-65880098, Email addresses: [zyzhong@suda.edu.cn](mailto:zyzhong@suda.edu.cn) (Z. Zhong); Tel/Fax: +86-512-65882060, Email addresses: [fhmeng@suda.edu.cn](mailto:fhmeng@suda.edu.cn) (F. Meng)

1. **Materials**

Paclitaxel (PTX, >98%, Shanghai Jinhe Bio-pharmaceutical Co.,ltd), ATN peptide (Ac-PhScNK-NH_2_, 98%, ChinaPeptides, Shanghai), PEG350 (Sigma-Aldrich) were used as received. CpG (CpG ODN 2018) and N-[[P(S)]-2'-deoxy-2',2'-difluoro-P-phenyl-5'-cytidine]-L-alanine benzyl ester (HPG, BR61501) were kindly donated by Genepharma Co., Ltd. and Brightgene Bio-Medical Technology Co., Ltd., respectively. PEG-P(CL-DTC) (*M*_n_ = 2.0-(1.1-0.9) kg/mol), ATN-PEG-P(CL-DTC) (*M*_n_ = 3.4-(1.1-1.1) kg/mol) and PEG-P(TMC-DTC)-sp (*M*_n_ = 5.0-(17.0-2.0)-0.2 kg/mol) were synthesized as our previous reports [1, 2].

Proteins were purchased and used as instruction, including granulocyte-macrophage colony stimulating factor (GM-CSF, PeproTech, 315-03), and antibodies like αCRT (Abcam, ab2907), Alexa Fluor® 647 goat anti-rabbit IgG (H&L) (Invitrogen, A21235), PerpCy5.5-αCD45 (Biolegend, 103132), FITC-αCD11c (Biolegend, 117306), APC-αCD80 (ebioscience, 17-0801-82), PE-αCD86 (ebioscience, 12-0861-83), APC-αCD3 (Biolegend, 100236), FITC-αCD3 (Biolegend, 100204), FITC-αCD8 (Biolegend, 100706), PE-αCD4 (Biolegend, 100408), Alexa 647-αFoxp3 (Biolegend, 320014), FITC-αCD11b (Biolegend, 101206), Alexa 647-αCD206 (Biolegend, 141711), PE-αF4/80 (123110), and PE/Cy7-αGr-1 (Biolegend, 108416). Assay kits were used according to supplier’s protocol, including micro BCA protein assay kit (Thermo Scientific, USA), annexin V-APC/7-AAD apoptosis kit (Multi sciences, AT105-100), cell cycle assay kit (Fcmacs), ATP assay kit (Beyotime, S0027), and ELISA kits including tumor necrosis factor alpha (TNF-α, Invitrogen, 88-7324-86), interferon gamma (TNF-α, Invitrogen, 88-8314-86), interleukin 10 (IL-10, Invitrogen, 88-7105-86).

4T1 cells were bought from the Type Culture Collection of the Chinese Academy of Sciences (Shanghai, China). Primary bone marrow dendritic cells (BMDCs) were from healthy Balb/c mice (5-6 weeks, female, 18-20 g, Charles River) as reported [3].

All animal experiments were approved by the Animal Care and Use Committee of Soochow University (P.R. China), and all protocols conformed to the Guide for the Care and Use of Laboratory Animals.

1. **Characterizations**

^1^H NMR spectra of copolymers were measured on an NMR spectrometer (DirectDrive2, Varian) with superconducting magnet at 600 MHz using DMSO-d_6_ and CDCl_3_ as solvent. Chemical shifts were calibrated in parts per million (ppm) referenced with respect to residual solvent (DMSO: δ 2.50, CHCl_3_: δ 7.26). The size and size distribution of the micelles were measured by a Zetasizer Nano-ZS (Malvern Instruments) using dynamic light scattering technique (DLS) at 25 ºC equipped with a 633 nm He-Ne laser using back scattering detection. The cross-linking of micelles was characterized by a UV-Vis Spectrophotometer (UH5300, HITACHI). The concentrations of HPG and PTX were measured by high performance liquid chromatography (HPLC, Waters 2690/5) using a C18 reversed-phase column (4.6×250 mm, 5 μm) (mobile phase: acetonitrile/H_2_O (v/v, 50/50), 1 mL/min, and UV 220 nm). The loading content (DLC) of CpG were determined by NanoDrop (NanoDrop 2000, Thermo). Flow cytometry (BD, FACS) was used to analyze the cell apoptosis and cell cycle arrest, expression of CRT on cell surface, the maturation of BMDCs as well as the infiltration of immune cells in tumor, spleen and lymph node. The fluorescence images of cells and tumor tissues were taken by a confocal laser scanning microscope (CLSM, TCS SP5, Leica). ELISA assays were quantified by measuring the absorbance at a wavelength of 450 nm using a microplate reader (Thermo Scientific Varioskan LUX).

1. **Characterization and drug release study of mG/P and ATN-mG/P**

mG/P and ATN-mG/P were prepared using nanoprecipitation method using PEG350 as a solvent. The size and size distribution of mG/P and ATN-mG/P were determined by DLS. The drug loading efficiency (DLE) and drug loading content (DLC) of mG/P and ATN-mG/P were determined by HPLC and calculated based on calibration curves. To achieve this, 0.2 mL mG/P or ATN-mG/P was dissolved in 0.8 mL methanol/ acetonitrile (v/v, 1/1). After 24 h, the solution was filtered and the supernatant was subject to HPLC measurement using acetonitrile/H_2_O (v/v, 50/50) as mobile phase, which yielded the elution time of HPG and PTX of 9.8 and 4.7 min, respectively. The standard curves were obtained from HPLC measurements of the solutions of HPG and PTX of known concentrations. Based on the standard curves, DLE and DLC were calculated using the following formula:

$$\text{DLC }\left( \text{wt.}\text{\%} \right)\text{ = }\frac{\text{Weight of loaded drugs in micelles}}{\text{Total weight of polymer and loaded drugs}}\text{ ×}\text{ }\text{100}$$

$$\text{DLE }\left( \text{\%} \right)\text{ = }\frac{\text{Weight of loaded drugs}}{\text{Weight of drugs in feed}}\text{ × 100}$$

The stability of mG/P in PB containing 10% fetal bovine serum or 10 mM glutathione (GSH) was monitored using DLS by tracking the size and size distribution. The UV absorbance of mG/P and PEG-P(CL-DTC) solution was measured at the same polymer concentration of 1.0 mg/mL.

To study the enzymatic degradation by cytidine deaminase (CDA) in the presence of serum, free HPG, mGem and mG/P (172 μM GEM equiv.) was incubated in 1 mL PB containing CDA (0.5 μg/mL) with or without 10% FBS at 37 ℃ for 4 h. Samples incubating with only PB or with PB containing a CDA inhibitor, tetrahydrouridine dihydrate (THU, 344 μM) and CDA were used as controls. After 4 h incubation, the solutions were centrifuged at 8000 rpm for 5 min, and the concentration of HPG in the supernatant was measured using HPLC (n = 3).

To investigate *in vitro* HPG and PTX release from mG/P under reductive and acidic conditions, 1 mL of mG/P (1 mg/mL) was transferred into dialysis bags (MWCO 14 kDa) and dialyzed against 25 mL PB (pH 7.4, 10 mM), PB (pH 7.4, 10 mM) with 10 mM GSH, and sodium acetate-acetic acid buffer solution (pH 5.0, 5 mM) under 200 rpm stirring at 37 ºC (n = 3). At predetermined time points, 7 mL release medium was taken for HPLC measurements and 7 mL fresh medium was added. The release medium was freeze-dried and redissolved in 500 μL a mixture of methanol and acetonitrile (v/v, 1/1), followed by quantification by HPLC (n = 3). The HPG release from mGem was studied similarly.

1. **Preparation and characterization of NanoCpG**

Polymersomal CpG (NanoCpG) was prepared as reported [1]. Briefly, 100 μL of PEG-P(TMC-DTC)-sp solution (DMF, 10 mg/mL) was added to 900 μL HEPES (5 mM, pH 6.8) containing CpG (100 μg) under stirring for 10 min. The solution was dialyzed twice in HEPES, once in PB/ HEPES (v/v, 1/1) and twice in PB. The size and size distribution of NanoCpG was measured by DLS and the CpG loading content by Nanodrop.

1. **Cell apoptosis and cell cycle arrest studies of mG/P and ATN-mG/P**

For cell apoptosis study, 4T1-luc cells were cultured in a 12-well plate (1×10^5^/well) with ATN-mG/P (Gem/PTX: 10/1), mG/P (Gem/PTX: 20/1, 10/1 or 5/1), mGem, and mPTX for 48 h (HPG: 0.5 μg/mL, PTX: 0.15 μg/mL, n = 3) using PBS as control. Then the cells were digested with trypsin (2.5 mg/mL), centrifuged (1000 rpm, 3 min), washed (2×, PBS) and resuspended in binding buffer. The cells were stained with Annexin V-APC and 7-AAD according to manual, and measured using flow cytometry and analyzed using FlowJo_V10. The cells stained with Annexin V-APC or 7-AAD only were used for determining gate for early or late apoptosis.

For cell cycle study, 4T1-luc cells were cultured 48 h with ATN-mG/P, mG/P, mGem and mPTX (HPG: 0.1 μg/mL, PTX: 0.03 μg/mL, n = 3). The cells were digested, washed, and fixed to 95% ethanol (4 mL) on ice. After 24 h storage at 4 °C, cell dispersions were treated by PI in binding buffer containing RNase A for 30 min. Then the proportion of cells at different phase of cell cycle was measured by flow cytometry and quantified using Modfit.

**Figure S1.** Characterization of mG/P. (a) Size distribution of mG/P at Gem/PTX molar ratio of 20/1, 10/1 or 5/1. (b) Size distribution of mGem and HPG solution in PEG350. (c) Long term stability mG/P before and after dialysis at room temperature. (d) Size distribution of mG/P in PB containing 10% FBS for 24 h. (e) HPG release from mGem with or without 10 mM GSH at pH 7.4 and 37 ℃ (n = 3).

**Table S1** Combination index (CI) of HPG and PTX in mG/P toward 4T1 cells

| Nanoparticles | Gem/PTX | HPG IC_50_ (μM) | PTX IC_50_ (μM) | CI |
| --- | --- | --- | --- | --- |
| mGem | - | 3.8 | - | - |
| mPTX | - | - | 1.4 | - |
| mG/P | 20/1 | 2.3 | 0.10 | 0.67 |
|  | 10/1 | 0.4 | 0.07 | 0.16 |
|  | 5/1 | 1.8 | 0.16 | 0.59 |

**Figure S2.** Cell cycle arrest of 4T1 cells at 48 h incubation with ATN-mG/P, mG/P (at Gem/PTX: 20/1, 10/1 or 5/1), and mGem at HPG dose of 0.1 μg/mL (0.34 μM). mPTX (PTX: 0.03 μg/mL) or PBS were used as contrlols.

**Figure S3**. The intact HPG contents of free HPG, mGem and mG/P at 4 h incubation with CDA with and without 10% serum (n = 3). Samples incubating with only PB or with PB containing THU (a CDA inhibitor, 344 μM) and CDA were used as controls.

**Figure S4.** The maturation of BMDCs co-cultured with 4T1 tumor cells after stimulation with mG/P and ATN-mG/P (n = 3). (a) Experimental design. Representative flow cytometric analysis and proportion of CD80^+^CD86^+^ mDCs of (b) mG/P (Gem/PTX: 20/1, 10/1 or 5/1) and (c) ATN-mG/P (Gem/PTX of 10/1). Dose: HPG: 1 μg/mL (1.7 μM), PTX: 0.3 μg/mL (0.34 μM).

**Figure S5**. Size changes (A) and HPG release (B) of mG/P in PB containing 0.01, 0.1 or 10 mM GSH over time.

**Figure S6.** BMDC maturation stimulated by ATN-mG/P (Gem/PTX= 10/1) combined with NanoCpG (n = 3). Representative flow cytometric analysis and proportions of mature BMDCs (CD11c^+^CD80^+^CD86^+^ mDCs). HPG: 1 μg/mL (1.7 μM), CpG: 0.4 μg/mL. *p < 0.05, ****p <0.0001.


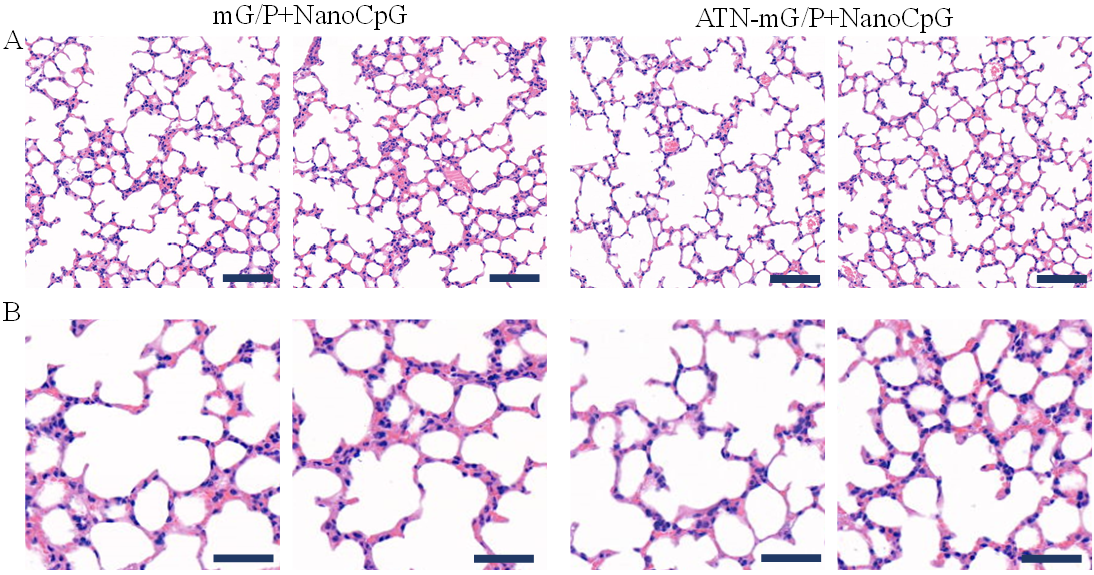


**Figure S7**. Representative H&E images of the lung slices of mice treated with mG/P+NanoCpG or ATN-mG/P+NanoCpG at two magnifications. Scale bars in A: 100 μm, B: 50 μm.

**References**

[1] J. J. Wei, D. Wu, S. S. Zhao, Y. Shao, Y. F. Xia, D. W. Ni, X. Y. Qiu, J. P. Zhang, J. Chen, F. H. Meng, Z. Y. Zhong, Immunotherapy of malignant glioma by noninvasive administration of TLR9 agonist CpG nano-immunoadjuvant, Adv. Sci. 9 (2022) 2103689. <https://doi.org/10.1002/advs.202103689>.

[2] X. Y. Qiu, Y. Qu, B. B. Guo, H. Zheng, F. H. Meng, Z. Y. Zhong, Micellar paclitaxel boosts ICD and chemo-immunotherapy of metastatic triple negative breast cancer, J. Control. Release 341 (2022) 498-510. <https://doi.org/10.1016/j.jconrel.2021.12.002>.

[3] H. Zheng, B. B. Guo, X. Y. Qiu, Y. F. Xia, Y. Qu, L. Cheng, F. H. Meng, Z. Y. Zhong, Polymersome-mediated cytosolic delivery of cyclic dinucleotide STING agonist enhances tumor immunotherapy, Bioactive Materials 16 (2022) 1-11. <https://doi.org/10.1016/j.bioactmat.2022.02.029>.
